# Supplementary material for: Causal Relationship between Aging and Anorexia Nervosa: A White-Matter-Microstructure-Mediated Mendelian Randomization Analysis
Source: Biomedicines. 2024 Aug 16;12(8):1874. doi: 10.3390/biomedicines12081874 (PMC11351342; doi:10.3390/biomedicines12081874)
Supplement: Supplementary file 1 [file biomedicines-12-01874-s001.zip › Supplementary Figure/Figure S4/Singe SNP plot of MR analysis for TL to ubm-b-2026.pdf]

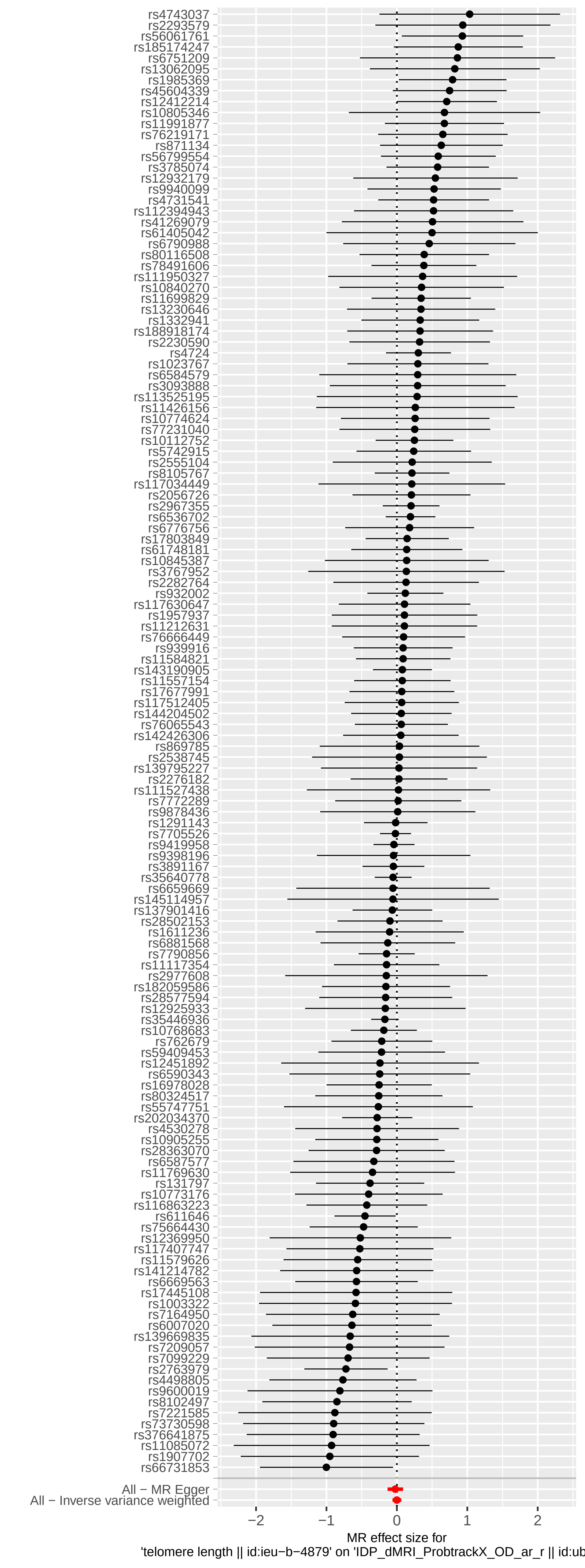
rs4743037
rs2293579
rs56061761
rs185174247
rs6751209
rs13062095
rs1985369
rs45604339
rs12412214
rs10805346
rs11991877
rs76219171
rs871134
rs56799554
rs3785074
rs12932179
rs9940099
rs4731541
rs112394943
rs41269079
rs61405042
rs6790988
rs80116508
rs78491606
rs111950327
rs10840270
rs11699829
rs13230646
rs1332941
rs188918174
rs2230590
rs4724
rs1023767
rs6584579
rs3093888
rs113525195
rs11426156
rs10774624
rs77231040
rs10112752
rs5742915
rs2555104
rs8105767
rs117034449
rs2056726
rs2967355
rs6536702
rs6776756
rs17803849
rs61748181
rs10845387
rs3767952
rs2282764
rs932002
rs117630647
rs1957937
rs11212631
rs76666449
rs939916
rs11584821
rs143190905
rs11557154
rs17677991
rs117512405
rs144204502
rs76065543
rs142426306
rs869785
rs2538745
rs139795227
rs2276182
rs111527438
rs7772289
rs9878436
rs1291143
rs7705526
rs9419958
rs9398196
rs3891167
rs35640778
rs6659669
rs145114957
rs137901416
rs28502153
rs1611236
rs6881568
rs7790856
rs11117354
rs2977608
rs182059586
rs28577594
rs12925933
rs35446936
rs10768683
rs762679
rs59409453
rs12451892
rs6590343
rs16978028
rs80324517
rs55747751
rs202034370
rs4530278
rs10905255
rs28363070
rs6587577
rs11769630
rs131797
rs10773176
rs116863223
rs611646
rs75664430
rs12369950
rs117407747
rs11579626
rs141214782
rs6669563
rs17445108
rs1003322
rs7164950
rs6007020
rs139669835
rs7209057
rs7099229
rs2763979
rs4498805
rs9600019
rs8102497
rs7221585
rs73730598
rs376641875
rs11085072
rs1907702
rs66731853
All − MR Egger
All − Inverse variance weighted
−2
−1
0
1
2
MR effect size for
'telomere length || id:ieu−b−4879' on 'IDP_dMRI_ProbtrackX_OD_ar_r || id:ub
